# Supplementary material for: The impact of work values on the professional development of primary and secondary school teachers: A moderated mediation model
Source: PLoS One. 2024 Nov 7;19(11):e0310078. doi: 10.1371/journal.pone.0310078 (PMC11542874; doi:10.1371/journal.pone.0310078)
Supplement: S1 File — (DOCX) [file pone.0310078.s002.docx]

STROBE Statement—checklist of items that should be included in reports of observational studies

|  | | | | Item No. | | Recommendation | Page  No. | Relevant text from manuscript |
| --- | --- | --- | --- | --- | --- | --- | --- | --- |
| **Title and abstract** | | | | 1 | | (*a*) Indicate the study’s design with a commonly used term in the title or the abstract | 1 | A moderated mediation model |
|  |  |  |  |  |  | (*b*) Provide in the abstract an informative and balanced summary of what was done and what was found | 2 | In total 873 primary and secondary school teachers were recruited at random; work values positively predicted professional development; work engagement partially mediated the relationship between work values and professional development, with the mediating effect accounted for 54.62% of the total effect; perceived organizational support regulated the direct path and the first half path of this mediation process. Specifically, compared with low organizational support, high organizational support can enhance the positive predictive effect of work values on work engagement and professional development |
| Introduction | | | | | | | |  |
| Background/rationale | | | | 2 | | Explain the scientific background and rationale for the investigation being reported | 2-5 | The motivation of Chinese teachers to participate in professional development was low, with only 21.02% of them participating; many countries have undertaken professional development activities that are generally planned and implemented by external agents to address problems and challenges. However, these activities have failed to significantly affect the practices of teachers; it is of great significance to explore the promoting factors promoting the professional development of primary and secondary school teachers under this background |
| Objectives | | | | 3 | | State specific objectives, including any prespecified hypotheses | 1-8 | Explore the relationship between work values and primary and secondary school teachers’ professional development, as well as the mediating role of work engagement and the moderating role of perceived organizational support;  **H1:** Work values can significantly positively predict the professional development of primary and secondary school teachers;  **H2:** Work engagement plays an mediating role between work values and the professional development of primary and secondary school teachers;  **H3:** Perceived organizational support not only moderates the direct prediction of work values of professional development, but also moderates the prediction of work values on work engagement |
| Methods | | | | | | | |  |
| Study design | | | | 4 | | Present key elements of study design early in the paper | 8-12 | Cross-sectional data collected; participants (primary and secondary school teachers); survey method and questionnaire tools; the relationship between variables; built a moderated mediation model |
| Setting | | | | 5 | | Describe the setting, locations, and relevant dates, including periods of recruitment, exposure, follow-up, and data collection | 8-11 | It is of great significance to explore the promoting factors promoting the professional development of primary and secondary school teachers under this background; Guangdong province in China; from August 1 to October 30, 2023 |
| Participants | | | | 6 | | (*a*) *Cohort study*—Give the eligibility criteria, and the sources and methods of selection of participants. Describe methods of follow-up  *Case-control study*—Give the eligibility criteria, and the sources and methods of case ascertainment and control selection. Give the rationale for the choice of cases and controls  *Cross-sectional study*—Give the eligibility criteria, and the sources and methods of selection of participants | 7 | Cross-sectional study—The convenience sampling method was employed to select students from colleges in Guangdong province of China to complete the questionnaires online from August 1 to October 30, 2023. In total, 873 valid questionnaires were obtained |
|  |  |  |  |  |  | (*b*) *Cohort study*—For matched studies, give matching criteria and number of exposed and unexposed  *Case-control study*—For matched studies, give matching criteria and the number of controls per case |  | NOTE: no Cohort study or Case-control study |
| Variables | | | | 7 | | Clearly define all outcomes, exposures, predictors, potential confounders, and effect modifiers. Give diagnostic criteria, if applicable | 2-6 | Not applicable. However, there are four main variables: perceived organizational support, work values, work engagement and professional development in this study |
| Data sources/ measurement | | | | 8* | | For each variable of interest, give sources of data and details of methods of assessment (measurement). Describe comparability of assessment methods if there is more than one group | 9 | In this study, 873 primary and secondary school teachers were selected by convenient sampling in Guangzhou, Shenzhen, Huizhou, Shaoguan and other places in Guangdong Province. A total of 912 questionnaires were obtained in the survey, 39 invalid questionnaires with missed key information, extreme response time or logical questions were eliminated (from August 1 to October 30, 2023). Finally 873 valid questionnaires were obtained (95.7% efficient). Among them, there are 217 male teachers (24.9%), 656 female teachers (75.1%), 189 teachers aged 25 years old and below (21.6%), 225 people aged 26-35 (25.8%), 216 teachers aged 36-45 (24.7%), 194 teachers aged 46-55 (22.2%), 49 over the age of 56 (5.60%); 270 teachers under 3 years (30.9%), 70 in 3-5 years (8.0%), 520 in 5-10 years (59.6%), 481 over 10 years (55.1%); 83 in junior colleges and below (9.5%), 748 undergraduates (85.7%), 42 master's degree and above (4.8%); 367 in primary school (42.0%), 326 in junior high school (37.3%), and 180 in senior high school (20.6%) |
| Bias | | | | 9 | | Describe any efforts to address potential sources of bias | 11 | The Harman single-factor test method was used to test the homologous deviation. The results showed that 14 factors with eigenvalues greater than 1 were obtained by unrotated principal component factor analysis, and the variance interpreted by the first factor was 14.889%, which was less than the critical standard of 40%. This indicated that there was no serious homologous bias in the research data |
| Study size | | | | 10 | | Explain how the study size was arrived at | 8-9 | The primary and secondary school teachers completed the four questionnaires online in Guangdong province in China |
| Quantitative variables | | 11 | | | Explain how quantitative variables were handled in the analyses. If applicable, describe which groupings were chosen and why | | 11 | SPSS 25.0 and Hayes macro PROCESS in SPSS software were used for all data analyses, and the mediation analyses were conducted by adopting PROCESS macro in the SPSS software (or the regression model) |
| Statistical methods | | 12 | | | (*a*) Describe all statistical methods, including those used to control for confounding | | 10-13 | SPSS 25.0 and Hayes macro PROCESS in SPSS software/multiple mediation analyses/Spearman’s rank correlation coefficients |
|  |  |  |  |  | (*b*) Describe any methods used to examine subgroups and interactions | | 10-13 | SPSS 25.0/Spearman’s rank correlation coefficients |
|  |  |  |  |  | (*c*) Explain how missing data were addressed | | 10-13 | The unqualified ones were excluded, and there are no missing data |
|  |  |  |  |  | (*d*) *Cohort study*—If applicable, explain how loss to follow-up was addressed  *Case-control study*—If applicable, explain how matching of cases and controls was addressed  *Cross-sectional study*—If applicable, describe analytical methods taking account of sampling strategy | | 7;10-13 | Cross-sectional study: the convenience sampling method was employed |
|  |  |  |  |  | (*e*) Describe any sensitivity analyses | |  | There are no sensitivity analyses in the study |
| Results | | | | | | | | |
| Participants | | 13* | | | (a) Report numbers of individuals at each stage of study—eg numbers potentially eligible, examined for eligibility, confirmed eligible, included in the study, completing follow-up, and analysed | | 9 | A total of 912 questionnaires were obtained in the survey, 39 invalid questionnaires with missed key information, extreme response time or logical questions were eliminated (from August 1 to October 30, 2023). Finally 873 valid questionnaires were obtained (95.7% efficient) |
|  |  |  |  |  | (b) Give reasons for non-participation at each stage | | 9 | The participants completed all the questionnaires and all the stages  The purpose of this study was explained, and all respondents provided informed consent electronically before starting without any payment. Their privacy and anonymity were guaranteed |
|  |  |  |  |  | (c) Consider use of a flow diagram | |  | NOTE: there is no need to use the flow diagram. |
| Descriptive data | | 14* | | | (a) Give characteristics of study participants (eg demographic, clinical, social) and information on exposures and potential confounders | | 9 | Among them, there are 217 male teachers (24.9%), 656 female teachers (75.1%), 189 teachers aged 25 years old and below (21.6%), 225 people aged 26-35 (25.8%), 216 teachers aged 36-45 (24.7%), 194 teachers aged 46-55 (22.2%), 49 over the age of 56 (5.60%); 270 teachers under 3 years (30.9%), 70 in 3-5 years (8.0%), 520 in 5-10 years (59.6%), 481 over 10 years (55.1%); 83 in junior colleges and below (9.5%), 748 undergraduates (85.7%), 42 master's degree and above (4.8%); 367 in primary school (42.0%), 326 in junior high school (37.3%), and 180 in senior high school (20.6%) |
|  |  |  |  |  | (b) Indicate number of participants with missing data for each variable of interest | | 7 | In total, 873 valid questionnaires were obtained with an effective rate of 95.70%, while 39 unqualified were excluded. |
|  |  |  |  |  | (c) *Cohort study*—Summarise follow-up time (eg, average and total amount) | |  | NOTE: not applicable |
| Outcome data | | 15* | | | *Cohort study*—Report numbers of outcome events or summary measures over time | |  | NOTE: not applicable |
|  |  |  |  |  | *Case-control study—*Report numbers in each exposure category, or summary measures of exposure | |  | NOTE: not applicable |
|  |  |  |  |  | *Cross-sectional study—*Report numbers of outcome events or summary measures | | 9-11 | 3 |
| Main results | | 16 | | | (*a*) Give unadjusted estimates and, if applicable, confounder-adjusted estimates and their precision (eg, 95% confidence interval). Make clear which confounders were adjusted for and why they were included | | 9 | NOTE: not applicable. However, we used 1000 bootstrap samples, and bias were corrected at a 95% confidence interval (CI) to calculate the indirect effect of each variable |
|  |  |  |  |  | (*b*) Report category boundaries when continuous variables were categorized | |  | NOTE: not applicable |
|  |  |  |  |  | (*c*) If relevant, consider translating estimates of relative risk into absolute risk for a meaningful time period | |  | Yes, if relevant, we will consider |
| Other analyses | 17 | | Report other analyses done—eg analyses of subgroups and interactions, and sensitivity analyses | | | | 8-11 | We reported the descriptive statistics, correlations statistics of major study variables,and the analysis of chain mediating effects of work values and teachers’ professional development |
| Discussion | | | | | | | | |
| Key results | 18 | | Summarise key results with reference to study objectives | | | | 17-20 | ① through relevant analysis, it is found that work values are positively correlated with the professional development of primary and secondary school teachers  ② the mediation analysis reveals that work values have a positive effect on work engagement, which in turn is positively associated with the professional development of primary and secondary school teachers  ③ perceived organizational support moderates the direct path and the first half path of this mediation process |
| Limitations | 19 | | Discuss limitations of the study, taking into account sources of potential bias or imprecision. Discuss both direction and magnitude of any potential bias | | | | 22 | One limitation of this study is that the convenience sample limits the universality of the results. This study uses the questionnaire survey method to explore the relationship between work values, work engagement, perceived organizational support and professional development of primary and secondary school teachers, which is a cross-sectional and not longitudinal study, and cannot well infer the causal association between the variables, which may limit the ability to draw causal inferences  Another limitation is that the sample size is limited to primary and secondary school teachers from several cities in Guangdong Province, which may restrict the generalizability of the results to other populations. |
| Interpretation | 20 | | Give a cautious overall interpretation of results considering objectives, limitations, multiplicity of analyses, results from similar studies, and other relevant evidence | | | | 22-23 | In this study, it is found that primary and secondary school teachers in China with high work values tend to have increased professional development through work engagement, especially those with a high perceived organizational support. The results indicate that perceived organizational support acts as a moderator between work values, work engagement, and professional development of primary and secondary school teachers |
| Generalisability | 21 | | Discuss the generalisability (external validity) of the study results | | | | 22-23 | This research sheds light on the antecedent factors of professional development and demonstrates that enhancing teachers’ perceived organizational support can lead to improvements in professional development  Overall, this study not only contributes to the existing literature by highlighting the importance of work engagement and perceived organizational support in the professional development among teachers with high work values, but also have important theoretical significance and practical value for improving the professional development of primary and secondary school teachers |
| Other information | | |  | | | | | |
| Funding | 22 | | Give the source of funding and the role of the funders for the present study and, if applicable, for the original study on which the present article is based | | | | 22 | Educational Science Planning Project (Higher Education Project; 2022GXJK383); Guangdong Sports Bureau Project (GDSS2022N143); Guangdong Higher Education Association Private higher education Professional Committee (2022MBGJ073); Guangdong Province Undergraduate College Teaching Quality and  Teaching Reform Project (2022J013/2022J038); Guangzhou Philosophy and Social Science Planning Project (2021GZGJ164); Guangzhou Xinhua University Science Research Project  (2020KYYB07) |

*Give information separately for cases and controls in case-control studies and, if applicable, for exposed and unexposed groups in cohort and cross-sectional studies.

**Note:** An Explanation and Elaboration article discusses each checklist item and gives methodological background and published examples of transparent reporting. The STROBE checklist is best used in conjunction with this article (freely available on the Web sites of PLoS Medicine at http://www.plosmedicine.org/, Annals of Internal Medicine at http://www.annals.org/, and Epidemiology at http://www.epidem.com/). Information on the STROBE Initiative is available at [www.strobe-statement.org.](http://www.strobe-statement.org.)
