# Supplementary material for: The impact of work values on the professional development of primary and secondary school teachers: A moderated mediation model
Source: PLoS One. 2024 Nov 7;19(11):e0310078. doi: 10.1371/journal.pone.0310078 (PMC11542874; doi:10.1371/journal.pone.0310078)
Supplement: Attachment — Submitted filename: Comments to Authors.docx [file pone.0310078.s003.docx]

**Comments to Authors**

**Abstract**

Overall, the abstract provides a concise overview of the study's objectives, methodology, key findings, and implications. Here are some critical comments for the authors:

1. Regarding the structure and alignment, the abstract would benefit from a more organized layout to enhance readability and coherence.
2. Ensuring that each sentence flows logically into the next and that key points are clearly emphasized could improve the overall structure.
3. Additionally, the abstract is somewhat lengthy and could be condensed to 200 words or less to maintain brevity while still effectively summarizing the study's objectives, methodology, findings, and implications.
4. Finally, while the abstract is generally well-written, attention to minor language and grammar issues, such as awkward phrasing or punctuation errors, is recommended to enhance clarity and professionalism. Addressing these aspects would help strengthen the abstract and make it more impactful for readers.

**Introduction**

1. The introduction is indeed well-written, providing a comprehensive overview of the research topic and its significance. However, there are a couple of areas where the authors could improve:
2. While the content is strong, attention to layout details such as spacing and alignment would enhance the presentation quality. Ensuring consistent formatting throughout the text will contribute to a polished appearance.
3. The authors should review the reference style to align with the guidelines of PLOS ONE. Consistency in referencing style is crucial for professionalism and adherence to journal requirements.
4. Although the introduction effectively identifies the gap in the literature, the specific research objectives could be articulated more clearly to guide readers on what to expect from the study. Clearly stating the research questions or hypotheses would provide readers with a roadmap for understanding the study's aims and contributions.
5. While the conclusion of the introduction emphasizes the significance of the study, the authors could further elaborate on the unique contributions of the research and how it advances knowledge in the field. Providing a more detailed discussion of the potential theoretical and practical implications of the study's findings would enhance the clarity and impact of the introduction.

**Literature Review**

1. Ensure that the referencing style aligns with the guidelines of the target journal, such as PLOS ONE. Consistency in referencing style is crucial for maintaining professionalism and meeting journal requirements.
2. Incorporate more updated references to ensure the literature review reflects the most current research in the field. Including recent studies will enhance the relevance and credibility of the literature review.
3. Clarify and separate the hypotheses related to the direct relationship between the independent variable (work values) and the mediator (work engagement), as well as the moderation effects of perceived organizational support. Presenting these hypotheses separately would improve clarity and facilitate understanding for the reader.
4. Provide a brief theoretical background before presenting the research model. This background should succinctly summarize relevant theoretical frameworks or concepts that support the study's hypotheses and research model. This addition will enhance the theoretical foundation of the study and provide context for the research model.

**Methodology**

1. The methodology section appears to prioritize demographic details over discussing the actual study methodology. It is recommended to provide a brief discussion of the demographic characteristics within the text and present the detailed demographic information in a table for better readability and organization.
2. The methodology does not address how data were collected from multiple provinces. If the data were collected online, it raises questions about the high response rate of 95.7%, which is unusually high for online surveys. Authors should provide justification for this high response rate to ensure the reliability and validity of the collected data.
3. The methodology discusses the measurement scales in detail. However, it would be more effective to consolidate this information into a single paragraph for clarity. Additionally, the discussion of reliability measures such as Cronbach's alpha and confirmatory factor analysis (CFA) should be moved to the results section, and the results should be presented in tabular format for easier reference.
4. As the authors is using control variables in the study also, I would suggest to provide brief detail about your control variables along with justification for using these variables as control.
5. The track changes should be removed from the manuscript, particularly from lines 191 to 193, to ensure the document's professional presentation.

**Results**

1. The results section is commendably well-written and presented, demonstrating a strong presentation of findings. However, one aspect that requires attention is the discussion of scale reliability and validity. It is essential to include a detailed discussion of these aspects within the text of the results section, along with presenting the corresponding statistics in a table format. This will ensure a comprehensive evaluation of the measurement instruments used in the study and enhance the transparency of the findings.

**Discussion**

2. The discussion is again well written however I would suggest, Provide a more thorough integration of current results with previous research on work values, work engagement, and organizational support in the context of teacher professional development.
3. Elaborate on specific strategies or interventions based on the findings and suggest future research directions to address remaining questions or potential limitations.

**Language Quality**

1. The paper is generally well-written and clear in its presentation. I observed few complex and long sentences which is suggested simplifying complex sentences to improve papers quality.
2. A thorough proofreading for grammatical accuracy and consistency in terminology would enhance the paper's overall quality.

**Comments to the Editor**

The study addresses an important topic, but improvements in formatting, referencing style consistency, and clarity of research objectives are needed. Streamlining demographic details and separating discussions on scale reliability and validity would enhance clarity. Additionally, more consideration of generalizability and causal relationships would strengthen the study. With minor revisions, particularly in language polishing, methodological justification, and practical implications, this paper has the potential to be a strong publication.

**Recommendation**

I recommend accepting this paper with minor revisions as outlined above.
